# Epigenetic Regulation of the NET Formation–Blood–Brain Barrier Axis in Ischemic Stroke: Mechanisms, Therapeutic Targets and Translational Perspectives

**DOI:** 10.3390/neurolint18060114

**Published:** 2026-06-08

**Authors:** Kirti Sharma, Baani Singh, Sarabjit Mastana, Monica Singh, Puneetpal Singh

**Affiliations:** 1 Division of Molecular Genetics, Department of Human Genetics, Punjabi University, Patiala 147002, India; kirti_rs22@pbi.ac.in (K.S.); baani_rs22@pbi.ac.in (B.S.); singhmonica2017@gmail.com (M.S.); 2 School of Sport, Exercise and Health Sciences, Loughborough University, Loughborough LE11 3TU, UK; s.s.mastana@lboro.ac.uk

**Keywords:** neutrophil extracellular traps, NETosis, NET formation, blood–brain barrier dysfunction, epigenetic regulation of NETosis, NETs and BBB dysfunction, ischemic stroke, non-coding RNAs in NETosis, NETs as mediator of BBB integrity, NETs and tight junction disassembly, NETs and basement membrane degradation, NETs and neuroinflammation, epigenetics of BBB-resident cells

## Abstract

Ischemic stroke elicits a rapid and sustained innate immune response that critically contributes to blood–brain barrier (BBB) breakdown and secondary neuronal injury. Among the cellular mediators involved, neutrophil extracellular traps (NETs) have emerged as potent effectors of neurovascular damage. However, the regulatory mechanisms governing NET formation and their prolonged impact on BBB integrity remain incompletely understood. Increasing evidence indicates that NET formation is an epigenetically regulated process, requiring chromatin remodeling, histone modifications, DNA methylation changes and non-coding RNA-mediated control within neutrophils under ischemic conditions. These epigenetic events license the extrusion of DNA–histone–enzyme complexes that directly injure endothelial cells, degrade tight junction proteins, activate innate immune signaling pathways and amplify neuroinflammatory cascades at the neurovascular unit. Moreover, NET-derived chromatin and associated mediators can induce transcriptional and epigenetic alterations in BBB cells, thereby sustaining barrier permeability and impairing vascular repair mechanisms. In this review, we synthesize current knowledge on the epigenetic regulation of NET formation and delineate how epigenetically regulated NETs function as key disruptors of BBB integrity in ischemic stroke. Understanding this NETosis–epigenetics–BBB axis may uncover novel therapeutic strategies aimed at preserving neurovascular integrity and limiting post-stroke brain injury.

## 1. Introduction

Energy crisis during ischemic stroke deprives the brain parenchyma of oxygen and glucose, leading to sequential events of ion imbalance, oxidative stress, neuroinflammation, and glutamate excitotoxicity, resulting in blood–brain barrier (BBB) disruption. BBB disruption is considerably prevalent in acute ischemic stroke (AIS), which allows infiltration of fluid, leading to vasogenic edema [1]. It increases intracranial pressure (ICP), which leads to secondary injury, malignant edema, brain herniation and hemorrhagic conversion, resulting in coma and death. BBB disruption is an independent predictor of hemorrhagic-driven mortality after thrombolytic and endovascular therapy [2]. Post-stroke BBB disruption is predictive of poor functional outcome irrespective of stroke severity and size, with abnormal neuronal firing leading to seizures and post-stroke epilepsy [3,4]. Moreover, BBB leakage is a prognostic marker of infarction severity in follow-up imaging [5]. Despite growing recognition of BBB disruption as a therapeutic target after cerebral injury, no approved intervention currently addresses BBB restoration directly, although multiple pharmacological and biological strategies have shown promise in preclinical and translational studies [6]. The in-depth understanding of BBB disruption may help to provide targeted therapy and drug delivery for salvaging ischemic tissue and post-stroke recovery.

Compromised BBB integrity is central to the post-stroke repair, recovery and remodeling of infarcted tissue [7]. Inflammation-driven activation of cytokines and proteolytic mediators triggers tight junction disassembly and basement membrane degradation. This BBB permeability allows peripheral immune cells to enter and localize onto the brain parenchyma, converting vascular inflammation into self-perpetuating neuroinflammation by activating microglia and astrocytes [7]. After stimulation from inflammatory signals released by cytokines, danger-associated molecular patterns (DAMPs) and activated platelets, neutrophils are the first to respond and infiltrate after BBB disruption. Highly prevalent during the first and third days after stroke onset, they move to infarcted tissue and make neutrophil extracellular traps (NETs). NETs are made up of DNA, proteases and histones. Histones are citrullinated by an enzyme, Peptidyl Deiminase-4 (PADI4), to stimulate NETs to decondense their nuclear chromatin. Chromatin in this loosened state composed of antimicrobial proteins like myeloperoxidase (MPO) and nuclear elastase (NE) is released into extracellular spaces by a process known as NETosis. These NETs induce endothelial cytotoxicity and dismantle tight junctions and basement membrane architecture, causing BBB dysfunction [7]. The terminology surrounding “NETosis” remains controversial, as the term has historically been used interchangeably with “NET formation” despite the recognition that extracellular trap release may occur through both lytic (suicidal) and non-lytic (vital) mechanisms. Because the suffix “-osis” conventionally denotes a cell death process, its universal application to all forms of NET release may introduce conceptual ambiguity. To improve mechanistic clarity, the present review preferentially uses the term “NET formation” as a broader descriptor, while reserving “NETosis” specifically for lytic or neutrophil death-associated NET release pathways.

NET formation is an epigenetically regulated process governed by individual or coordinated activation of DNA methylation, histone modifications, nucleosome remodeling, chromatin reorganization, and miRNAs, lncRNAs and RNA editing [8]. The NET epigenetic gene expression link influences several phases of cerebral ischemia, resulting in post-stroke repair, remodeling and recovery. Epigenetic modulation, through these processes, impacts chromatin decondensation, reactive oxygen species generation and protease activity, thereby determining the magnitude and pathogenic potential of NET formation in cerebrovascular inflammation [9]. However, a systematic mechanistic link between NET-associated epigenetic regulation and BBB disruption remains unexplored. Elucidating this epigenetic regulation may help explain inter-individual susceptibility to NET-mediated BBB failure following ischemic stroke.

This review aims to critically examine emerging evidence linking NET formation with epigenetic regulation and BBB disruption in ischemic stroke. We aim to integrate current knowledge on the molecular mechanisms governing NET formation, with particular emphasis on chromatin remodeling, histone modifications, DNA methylation and non-coding RNA-mediated control and to delineate how these processes contribute to sustained BBB permeability and neurovascular injury. By synthesizing findings from experimental and translational studies, this review seeks to highlight NETs as epigenetically regulated effectors of BBB dysfunction and to identify key knowledge gaps that may guide future the therapeutic targeting of the neurovascular unit in ischemic stroke. Furthermore, we propose an integrated “epigenetic–immune–vascular axis” in which ischemia-induced inflammatory signaling primes neutrophils epigenetically for exaggerated NET formation, while NET-derived chromatin components sustain BBB dysfunction through secondary epigenetic alterations in BBB-resident cells. Based on this framework, we further hypothesize that NET-mediated BBB disruption may depend on synergistic interactions between DNMT-mediated transcriptional priming and PADI4-driven histone citrullination, thereby representing a testable epigenetic mechanism underlying sustained neurovascular dysfunction in ischemic stroke.

## 2. Methodology and Scope

The present study was conducted as a narrative review focusing on the mechanistic interplay of NET formation, BBB dysfunction, neurovascular inflammation and epigenetic regulation during ischemic stroke. This review aimed to synthesize current experimental, translational and clinical evidence describing the role of NET-associated inflammatory and thromboinflammatory pathways in ischemic brain injury.

A comprehensive literature search was performed using the PubMed, Scopus and Google Scholar databases for studies published in peer-reviewed journals in the English language up to May 2026. The search strategy utilized combinations of keywords and Medical Subject Headings (MeSH) terms including “ischemic stroke”, “cerebral ischemia”, “neutrophil extracellular traps”, “NET formation”, “NETosis”, “blood–brain barrier”, “neuroinflammation”, “epigenetics”, “PADI4”, “histone citrullination”, “oxidative stress”, “STING signaling”, “thromboinflammation” and “endothelial dysfunction”. Reference lists of selected articles were additionally screened to identify other relevant studies.

Original research articles, experimental studies, translational investigations and relevant review articles examining NET-associated mechanisms in ischemic stroke and BBB dysfunction were considered for inclusion. Particular emphasis was placed on studies describing endothelial injury, inflammatory signaling, chromatin remodeling, thromboinflammatory interactions and neurovascular dysfunction associated with NET formation. Preference was given to studies involving ischemic stroke models, cerebral endothelial systems and neurovascular unit-related investigations.

Studies lacking mechanistic relevance to NET-mediated neurovascular injury, articles with insufficient methodological clarity and non-peer-reviewed sources were excluded from detailed consideration. In areas where direct ischemic stroke-specific evidence was limited, supportive mechanistic findings from related inflammatory conditions including sepsis, cardiovascular disease, neurodegenerative disorders and systemic inflammatory models were incorporated cautiously to provide broader biological context and mechanistic interpretation.

## 3. Clinical and Translational Evidence Linking NETs to BBB Dysfunction in Ischemic Stroke

Accumulating evidence from human studies provides strong translational support for a clinically significant role of NETs in ischemic stroke pathobiology, linking thromboinflammation, BBB dysfunction and adverse neurological outcomes [7,10]. Circulating markers of NET formation, including MPO-DNA complexes, citrullinated histone H3 (CitH3), cell-free DNA and histone–DNA complexes, have been consistently reported to be elevated in patients with acute ischemic stroke [11,12]. These biomarkers not only reflect systemic neutrophil activation but also correlate with National Institutes of Health Stroke Scale scores, infarct volume, early neurological deterioration and poorer functional outcomes, supporting their emerging prognostic relevance [13]. Reduced endogenous DNase activity in stroke patients further suggests that impaired NET clearance may contribute to sustained immunothrombotic injury [12]. Details of clinical and translational evidence supporting NETs as mediators of BBB dysfunction in ischemic stroke are shown in Table 1.

Histopathological analyses of thrombi retrieved during mechanical thrombectomy have provided direct evidence that NETs are structural and functional components of human stroke thrombi [11,14]. NET-rich thrombi exhibit increased architectural complexity and resistance to fibrinolysis, partly due to extracellular DNA scaffolds and histone-associated proteins, which reduce susceptibility to tissue plasminogen activator-mediated clot dissolution [15,16,17]. These observations have important translational implications, particularly as adjunctive DNase I treatment has shown potential in experimental and ex vivo settings to enhance thrombolysis and improve clot dissolution [16,18]. Together, these studies position NETs as important mediators of impaired reperfusion and therapeutic resistance.

Clinical evidence also supports a close association between NET burden and BBB dysfunction. Elevated circulating NET markers correlate with imaging features of vascular permeability, including contrast extravasation, vasogenic edema and microvascular leakage, suggesting a link between NET activity and barrier disruption in human stroke [19,20]. Moreover, NET burden has been associated with increased risk of hemorrhagic transformation following reperfusion therapies, further implicating NET-mediated vascular instability in clinically significant BBB injury [21,22].

These findings are consistent with the established cytotoxic and proteolytic effects of extracellular histones, neutrophil elastase and other NET-associated mediators on endothelial integrity and tight junction architecture.

Beyond serving as biomarkers of vascular injury, NETs appear to actively participate in thromboinflammatory amplification in human stroke. Increased platelet High-Mobility Group Box 1 (HMGB1) signaling and platelet–neutrophil aggregates have been associated with enhanced NET formation, supporting a mechanism whereby platelet-driven immunothrombosis further reinforces NETosis and vascular injury [23,24]. This aligns with evidence that NET-associated proteases and histones contribute not only to thrombus stability and endothelial damage but also to impaired reperfusion and neurovascular dysfunction [22,25].

Although direct clinical evidence linking epigenetic regulation of NET formation to BBB dysfunction remains limited, emerging data suggest that epigenetic mechanisms may influence inter-individual variability in NET burden and stroke outcomes. Altered DNA methylation patterns and dysregulated non-coding RNAs, including miR-146a and miR-155, have been identified in circulating immune cells of stroke patients and are known to regulate inflammatory signaling pathways involved in neutrophil activation and NET formation [26,27]. These observations raise the possibility that epigenetic regulation contributes to patient-specific differences in thromboinflammation, BBB injury and therapeutic response.

Alluding to these, clinical and translational studies position NETs not merely as biomarkers but also as mechanistic drivers of stroke severity, thromboinflammation, impaired reperfusion and BBB dysfunction. These findings provide a strong rationale for targeting NET formation as both a biomarker axis and a therapeutic strategy in ischemic stroke while also highlighting epigenetic regulation as an emerging translational dimension that may inform precision approaches aimed at preserving neurovascular integrity and improving clinical outcomes.

Despite their translational potential, interpretation of circulating NET biomarkers remains challenging. Commonly used markers such as MPO-DNA complexes, citrullinated histones and cell-free DNA may not be entirely specific to NET formation and can also reflect systemic inflammation, thrombosis or non-specific tissue injury. Variability in detection methodologies, sample timing and biomarker stability further complicates standardization and clinical interpretation across stroke cohorts.

**Table 1:** Clinical and translational evidence supporting NETs as mediators of BBB dysfunction in ischemic stroke.

Category	Key Findings and Evidence	Clinical and Translational Significance	References
Systemic Biomarkers	Elevated MPO-DNA, CitH3 and cfDNA correlate with NIHSS scores, infarct volume and neurological deficits.	Useful for prognostic stratification and monitoring systemic disease activity.	[12,13,15,16,28]
Thrombus and Reperfusion	NET-rich thrombi in human clots increase structural complexity and tPA resistance, associated with mechanical thrombectomy failure.	Provides a rationale for DNase I as an adjunct to improve thrombolysis/recanalization.	[13,17,18,19,20,21,29]
Neurovascular Unit (NVU)	NET burden links to BBB permeability, vasogenic edema and hemorrhagic transformation via histone/protease-mediated injury.	Identifies NETs as a primary mediator of vascular instability and secondary brain injury.	[12,22,23,24]
Cellular Signaling	Platelet HMGB1 and platelet–neutrophil aggregates (PNAs) drive sustained NETosis and immunothrombosis.	Highlights upstream targets to prevent thromboinflammatory amplification.	[25,26]
Epigenetics and Clearance	Reduced endogenous DNase activity and dysregulates DNA methylation/microRNAs (miR-146a/155).	Supports precision medicine and epigenetic modulation as therapeutic avenues.	[14,27,30]

cfDNA: cell-free DNA; CitH3: citrullinated Histone 3; DNase I: deoxyribonuclease I; HMGB1: High-Mobility Group Box 1; mi-R: microRNA; MPO-DNA: Myeloperoxidase–DNA complex; NETs: neutrophil extracellular traps; NIHSS: National Institutes of Health Stroke Scales; NVU: neurovascular unit; PNAs: Peptide Nucleic Acid; tPA: tissue plasminogen activator.

## 4. Structure and Functional Organization of the Blood–Brain Barrier

The BBB is a highly specialized and dynamic interface that regulates molecular exchange between the systemic circulation and the central nervous system (CNS), thereby preserving neuronal homeostasis [30]. It is primarily formed by brain microvascular endothelial cells, which are structurally and functionally distinct from peripheral endothelial cells due to the presence of continuous tight junctions, minimal transcytosis and highly selective transport systems [28,30]. These endothelial cells constitute the core physical barrier, restricting paracellular diffusion and maintaining a controlled microenvironment essential to neuronal function [28].

Tight junctions are the principal structural determinants of BBB integrity and consist of transmembrane proteins such as claudin-5, occludin, and junctional adhesion molecules (JAMs), which are anchored to the cytoskeleton through adaptor proteins including zonula occludens-1 (ZO-1), ZO-2 and ZO-3 [29]. These complexes form a continuous intercellular seal that confers high electrical resistance and restricts the passage of ions and macromolecules. In addition to tight junctions, adherens junctions mediated by vascular endothelial cadherin (VE-cadherin) contribute to endothelial stability and intracellular signaling required for barrier maintenance [31].

**Figure 1:**
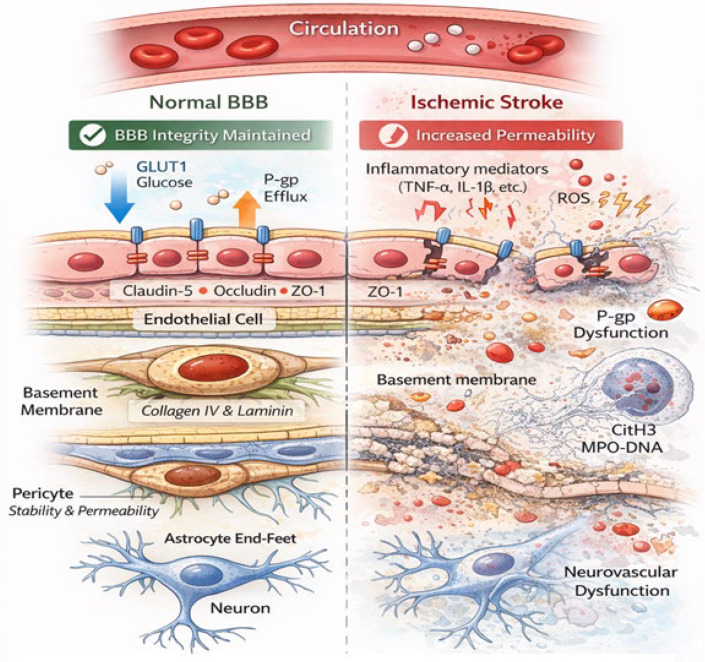
Structural and functional alterations of the blood–brain-barrier (BBB) under physiological and ischemic stroke conditions. Under physiological conditions (**left**), endothelial tight junctions (claudin-5, occludin, and ZO-1), intact basement membrane, and coordinated transport (GLUT1 influx and P-gp efflux) maintain BBB integrity within the neurovascular unit. During ischemic stroke (**right**), inflammatory mediators, reactive oxygen species (ROS), and proteolytic enzymes disrupt tight junctions, impair transporter function, and degrade the basement membrane, leading to increased permeability. Neutrophil extracellular traps (NETs), marked by citrullinated histone H3 (CitH3) and MPO–DNA complexes, further exacerbate endothelial injury and BBB breakdown, resulting in neurovascular dysfunction. GLUT1: glucose transporter type 1; P-gp: Permeability-glycoportein 1; TNF-α: tumor necrosis factor-alpha; IL-1β: interleukin-1 beta; ZO-1: zonula occludens-1.

The BBB is an integral component of the neurovascular unit (NVU), which comprises endothelial cells, pericytes, astrocytes, neurons and extracellular matrix components. Pericytes, embedded within the basement membrane, play a crucial role in regulating endothelial cell proliferation, vascular stability and permeability (Figure 1). Loss or dysfunction of pericytes has been strongly associated with increased BBB permeability and vascular leakage [32]. Astrocytic end-feet ensheathe the cerebral microvasculature and secrete regulatory factors such as angiopoietin-1, glial-derived neurotrophic factor and transforming growth factor-β, which promote tight junction integrity and endothelial differentiation [33].

The basement membrane provides structural and biochemical support to the BBB and is composed of type IV collagen, laminin, nidogen, and heparan sulfate proteoglycans such as perlecan [34]. This matrix stabilizes endothelial–pericyte interactions and serves as a signaling scaffold regulating cellular adhesion, migration and repair. Disruption of basement membrane integrity significantly compromises BBB function and facilitates immune cell infiltration.

Functionally, the BBB maintains strict control over molecular trafficking through specialized transport systems. Glucose transport is mediated by glucose transporter-1 (GLUT1), while amino acids are transported via selective carrier systems such as LAT1 [35]. Efflux transporters, including P-glycoprotein (P-gp) and breast cancer resistance protein (BCRP), actively remove xenobiotics and metabolic waste, thereby contributing to neuroprotection [36]. In addition, receptor-mediated transcytosis enables the selective passage of macromolecules such as insulin and transferrin across the barrier.

Under physiological conditions, these structural and functional components operate in a coordinated manner to maintain BBB integrity. However, during ischemic stroke, inflammatory mediators, oxidative stress and proteolytic enzymes disrupt tight junction complexes, alter transporter function and degrade basement membrane components, leading to increased permeability and neurovascular dysfunction [7,37]. Understanding the baseline architecture of the BBB is therefore essential to delineating how pathological mechanisms, including epigenetically regulated NET formation, compromise barrier integrity.

## 5. Temporal Dynamics of NET Formation and BBB Disruption in Ischemic Stroke

The temporal evolution of NET formation and its impact on BBB integrity represent a critical yet underexplored dimension of ischemic stroke pathophysiology. Emerging evidence indicates that NET formation is not a static process but follows a dynamic, phase-dependent trajectory that closely parallels the progression of neurovascular injury. Understanding these temporal patterns is essential to identifying optimal therapeutic windows and for delineating stage-specific mechanisms of BBB disruption.

In the early phase (within 0–6 h of ischemic onset), rapid activation of the innate immune response leads to recruitment and infiltration of circulating neutrophils into the ischemic brain microvasculature. This process is facilitated by endothelial activation, upregulation of adhesion molecules such as ICAM-1 and P-selectin and increased vascular permeability [38]. Initial NET formation can occur within this timeframe, triggered by DAMPs, platelet activation and complement signaling [39]. Although NET burden is relatively modest at this stage, NET components such as extracellular DNA and histones begin to exert cytotoxic effects on endothelial cells, initiating early tight junction destabilization and microvascular dysfunction [40].

During the subacute phase (approximately 1–3 days post-stroke), NET formation reaches its peak and plays a central role in amplifying BBB disruption [41]. This period is characterized by sustained neutrophil activation, elevated intracellular calcium levels and robust activation of PADI4, leading to extensive histone citrullination and chromatin decondensation [25]. Accumulation of NETs within the cerebral microvasculature promotes degradation of tight junction proteins such as claudin-5 and occludin through the action of neutrophil elastase and matrix metalloproteinases, while extracellular histones induce endothelial cytotoxicity and oxidative stress [42,43]. Concurrently, NET-mediated thromboinflammation contributes to microvascular occlusion and impaired reperfusion, further exacerbating ischemic injury. Clinically, this phase correlates with maximal BBB permeability, vasogenic edema formation and increased risk of hemorrhagic transformation [37,44].

In the chronic phase (days to weeks after stroke), although the acute inflammatory response begins to subside, the effects of NET formation persist through sustained epigenetic and transcriptional reprogramming within the neurovascular unit. NET-derived chromatin and associated mediators continue to interact with endothelial cells, astrocytes and microglia, inducing long-lasting changes in histone modification patterns, DNA methylation and inflammatory gene expression [45,46]. These epigenetic alterations contribute to prolonged BBB permeability, impaired tight junction reassembly and defective vascular repair mechanisms. In addition, persistent low-grade NET formation and residual extracellular DNA can maintain activation of innate immune signaling pathways such as TLR9 and cGAS–STING, thereby sustaining chronic neuroinflammation and delaying functional recovery [47,48]. Based on these studies, researchers have established a temporally orchestrated model in which NET formation evolves from an early initiator of endothelial injury to a dominant driver of BBB breakdown in the subacute phase and ultimately to a mediator of persistent neurovascular dysfunction through epigenetic imprinting in the chronic stage. This temporal framework underscores the importance of stage-specific therapeutic strategies. Early-phase interventions may focus on limiting neutrophil recruitment, endothelial activation and thromboinflammatory signaling, whereas the subacute phase may benefit from targeted inhibition of PADI4 activity, NET formation and protease-mediated BBB injury. In the chronic stage, modulation of persistent inflammatory and epigenetic pathways may help restore BBB integrity, promote vascular repair and improve neurological recovery following ischemic stroke.

## 6. Triggers of NET Formation During Ischemic Stroke

Following ischemic stroke, extensive cellular injury and metabolic stress lead to the release of DAMPs, including HMGB1, extracellular ATP, mitochondrial DNA, heat shock and S100 proteins, and necrotic and apoptotic signals, into the neurovascular microenvironment. These DAMPs are sensed by infiltrating neutrophils through pattern-recognition receptors such as Toll-like receptors (TLR2, TLR4 and TLR9), receptor for advanced glycation end products (RAGE) and purinergic receptors [49]. Engagement of these receptors initiates intracellular signaling cascades characterized by calcium influx, reactive oxygen species (ROS) generation and activation of kinase pathways, all of which contribute to the activation of PADI4 [50].

In addition to DAMPs, several other stroke-associated triggers contribute to PADI4 activation. Activated platelets represent a major source of NETosis-inducing signals by establishing direct physical and molecular interactions with neutrophils. Upon platelet activation, surface expression of P-selectin enables binding to P-selectin glycoprotein ligand-1 (PSGL-1) on neutrophils, facilitating platelet–neutrophil aggregation and close cellular contact. In parallel, platelets release soluble mediators such as platelet factor 4 (PF4/CXCL4), which binds to neutrophil surface receptors and amplifies intracellular signaling pathways associated with NET formation [51]. Platelets also secrete HMGB1, which engages pattern-recognition receptors on neutrophils, including TLR4 and RAGE, thereby acting as a damage-associated molecular signal. These platelet-derived signals converge by activating complementary calcium-entry pathways in neutrophils [52]. P-selectin-mediated cell–cell contact primes neutrophil signaling, while PF4 and HMGB1 engage chemokine and pattern-recognition receptors, triggering phospholipase–inositol triphosphates (PLC–IP_3_)-dependent calcium release from intracellular stores and calcium influx across the plasma membrane [53]. The resulting sustained rise in intracellular calcium provides the critical signal required for PADI4 activation and NET formation. Abundant presence of thrombin and fibrin, within ischemic and thrombotic vessels, further stimulate neutrophils via protease-activated receptors, promoting intracellular calcium elevation and chromatin remodeling. Complement activation products, particularly C3a and C5a (anaphylatoxins), also act as potent neutrophil activators that amplify ROS generation and facilitate PADI4-dependent chromatin decondensation [54].

Proinflammatory cytokines and chemokines released during cerebral ischemia, including tumor necrosis factor-α (TNF-α), interleukin-1β (IL-1β) and interleukin-8 (CXCL8), further prime neutrophils for NET formation by enhancing transcriptional activation, metabolic reprogramming and calcium sensitivity. Simultaneously, hypoxia and ischemia-induced metabolic stress activate hypoxia-inducible factor-1α (HIF-1α) signaling and mitochondrial dysfunction, leading to increased ROS production that synergizes with calcium-dependent PADI4 activation [55]. In certain contexts, microbial products and sterile pathogen-associated molecular pattern like ligands may further potentiate NETosis in patients with concurrent infections or systemic inflammation.

## 7. NET Formation, Epigenetic Regulation and BBB Dysfunction in Ischemic Stroke

NET formation, or NETosis, is a heterogeneous process based on underlying signaling pathways, cellular fate and regulatory mechanisms. In the context of ischemic stroke, these forms of NET formation are increasingly recognized as critical mediators linking epigenetic regulation to BBB dysfunction and neurovascular injury [56,57].

Suicidal or lytic NETosis represents the classical form of NET formation and is primarily driven by ROS-dependent signaling cascades. This process involves activation of NADPH oxidase (NOX2), followed by nuclear translocation of NE and MPO, culminating in chromatin decondensation and cell lysis [58]. A central epigenetic event in this pathway is the activation of PADI4, which catalyzes histone citrullination, leading to loss of chromatin compaction and enabling NET release [59,60].

In ischemic stroke, PADI4-mediated NET formation has been directly implicated in BBB disruption and impaired vascular remodeling. Experimental studies demonstrate that increased PADI4 expression enhances NET formation, which in turn exacerbates BBB breakdown and limits post-stroke neovascularization [7,25]. Conversely, pharmacological inhibition or genetic deletion of PADI4 reduces NET burden, improves vascular integrity and promotes functional recovery [61,62]. These findings highlight histone modification-driven NET formation as a key epigenetic mechanism contributing to neurovascular injury.

Vital or non-lytic NET formation is characterized by rapid extrusion of chromatin without immediate neutrophil death, allowing cells to retain functional activity. This form is often triggered by interactions with activated platelets, complement factors and DAMPs, which are abundantly released during cerebral ischemia [63]. Unlike suicidal NETosis, vital NET formation may proceed independently of NADPH oxidase activity and can involve vesicular export of nuclear or mitochondrial DNA [64].

In stroke, platelet–neutrophil interactions and DAMP signaling, particularly involving HMGB1, have been shown to induce NETosis and amplify vascular injury [23]. NETs generated through these pathways contribute to endothelial activation, microvascular occlusion and BBB permeability changes, thereby promoting thromboinflammation and worsening neurological outcomes [65]. The rapid kinetics of vital NETosis suggest its role in early-phase BBB disruption following ischemic insult.

Mechanistically, NET formation can also be categorized based on dependence on NADPH oxidase activity. NOX-dependent NETosis is associated with ROS generation and classical suicidal NETosis, whereas NOX-independent pathways rely on alternative sources of oxidative stress, including mitochondrial ROS and calcium signaling [66]. These pathways converge on epigenetic regulators such as PADI4, linking intracellular signaling to chromatin remodeling [66,67]. Although calcium influx, ROS generation and PADI4 activation are critical regulators of NET formation, these processes primarily represent upstream signaling and post-translational regulatory events rather than epigenetic mechanisms themselves. Their contribution to epigenetic regulation mainly arises through facilitation of chromatin decondensation, histone citrullination and downstream transcriptional remodeling associated with NET release. Accordingly, within the present conceptual framework, epigenetic regulation should be interpreted primarily as a modulatory and integrative mechanism interacting with inflammatory, oxidative and thromboinflammatory signaling pathways rather than as an isolated principal driver of BBB dysfunction.

In ischemic stroke, oxidative stress is a major driver of BBB dysfunction and ROS-mediated NET formation contributes to degradation of tight junction proteins and activation of proteolytic enzymes such as matrix metalloproteinases [37]. NET-associated proteases and histones directly damage endothelial cells and disrupt BBB integrity, facilitating immune cell infiltration and edema formation [43].

Regardless of the initiating pathway, all forms of NET formation converge on a common pathological outcome—BBB disruption. NET components, including extracellular DNA, histones and proteases, degrade tight junction proteins, alter endothelial permeability and promote a prothrombotic microenvironment. In experimental stroke models, NET accumulation correlates with increased BBB leakage, reduced vascular repair and worsened neurological outcomes [7]. Importantly, the therapeutic targeting of NETosis using DNase I or PADI4 inhibitors has been shown to restore BBB integrity and improve recovery, underscoring the translational significance of these pathways [68].

Different forms of NET formation, such as suicidal, vital, and NOX-dependent or -independent, represent mechanistically distinct yet functionally convergent processes that link epigenetic chromatin remodeling to BBB dysfunction in ischemic stroke. Understanding these interconnected pathways provides a critical framework for developing targeted interventions aimed at modulating NET formation and preserving neurovascular integrity. Nonetheless, important controversies remain regarding the precise contribution of different NET formation pathways to ischemic stroke pathology. While suicidal, vital and NOX-independent NET formation have all been implicated in neurovascular injury, their relative temporal and spatial contributions within the ischemic brain remain incompletely resolved. In addition, some studies suggest that NET deposition within ischemic tissue may be more restricted and context-dependent than previously assumed, highlighting ongoing uncertainty regarding the extent to which NETs uniformly drive BBB dysfunction across different stroke stages and microenvironments.

Despite substantial evidence supporting a pathogenic role for NETs in ischemic stroke, contradictory findings suggest that NET formation may exhibit context-dependent effects [56,69]. While multiple studies show PADI4-driven NET formation exacerbates thrombosis, BBB disruption and impaired vascular remodeling [7,70], some studies have questioned the extent of neutrophil infiltration and NET deposition within the ischemic brain parenchyma, suggesting that NET-associated injury may be spatially and temporally restricted rather than uniformly detrimental [71]. Moreover, because neutrophils also participate in debris clearance and early host defense, complete suppression of NET formation could potentially interfere with reparative immune responses [72]. These observations indicate that the impact of NET formation may depend on timing, stroke stage and degree of inflammatory activation, emphasizing the need for temporally selective therapeutic modulation rather than indiscriminate NET inhibition [73]. Although accumulating experimental evidence supports the involvement of NET-associated inflammatory mediators in BBB dysfunction, several mechanistic interactions remain incompletely characterized in the specific context of ischemic stroke, and some proposed pathways are currently inferred from related inflammatory and vascular disease models.

## 8. Epigenetic Regulation of NET Formation

**Figure 2:**
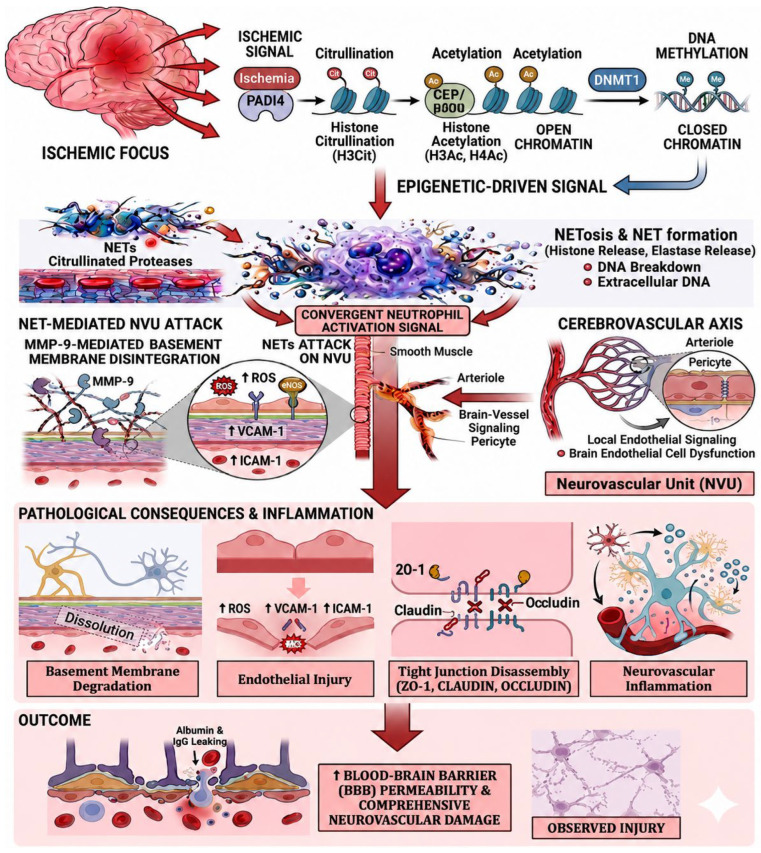
Epigenetic transformation and the resultant neurovascular unit (NVU) injury cascade in the pathogenesis of cerebral ischemia. Following the onset of an ischemic stroke, the Ischemic Focus triggers a complex molecular transition characterized by rapid epigenetic remodeling. This includes PADI4-mediated histone citrullination (H3Cit), CBP/p300-dependent histone acetylation (H3Ac and H4Ac), and DNMT1-mediated DNA methylation, which collectively modulate chromatin accessibility to favor a proinflammatory transcriptome. This epigenetic signature serves as a critical driver for NETosis, the release of neutrophil extracellular traps (NETs) laden with citrullinated proteases and elastase. These NETs directly attack the neurovascular unit (NVU), synergizing with MMP-9 to facilitate basement membrane disintegration and promoting endothelial dysfunction through the upregulation of reactive oxygen species (ROS) and adhesion molecules (VCAM-1 and ICAM-1). The structural integrity of the blood–brain barrier (BBB) is subsequently compromised via the disassembly of tight junction proteins (ZO-1, claudin, and occludin), leading to the paracellular leakage of macromolecules such as Albumin and IgG. This cascade culminates in comprehensive neurovascular inflammation and irreversible parenchymal damage, representing the core pathological outcome of ischemic injury. Ac: acetylation; H3Cit: citrullinated histone 3; CBP/p300: CREB-Binding Protein/E1A-binding protein p300 complex; DNMT1: DNA methyltransferase 1; H3Ac: acetylated histone 3; H4Ac: acetylated histone 4; ICAM-1: Intercellular Adhesion Molecule-1; MMP-9: matrix metalloproteinase-9; PADI4: Peptidyl Arginine Deiminase 4; ROS: reactive oxygen species; VCAM-1: Vascular Cell Adhesion Molecule-1; ZO-1: zonula occludens-1.

Epigenetic mechanisms critically govern neutrophil extracellular trap formation and determine its pathological impact on BBB integrity. Dynamic chromatin modifications, particularly histone citrullination, acetylation and methylation, permit nuclear decondensation required for NET release, while simultaneously reprogramming inflammatory gene expression in activated neutrophils [74]. The resulting NET components interact directly with cerebrovascular endothelium, promoting endothelial injury, tight junction destabilization and sustained BBB leakage. Thus, epigenetically driven NET formation represents a key molecular axis linking neutrophil activation to neurovascular damage under ischemic conditions (Figure 2).

### 8.1. Citrullination

Histone citrullination is an essential step for the formation of NETs and their release. This process is regulated by activation of PADI4 by different triggers in the cytoplasm and then its localization in the nucleus of neutrophils because it bears a nuclear localization signal (NLS) [60]. Inside the nucleus, PADI4 citrullinates H2A, H3 and H4 histones; specifically, citrullination of H3 (CitH3) is crucial for the formation of NETs, as it is a major component released during NETosis and a critical mediator of BBB disruption.

PADI4 is a calcium-dependent enzyme that catalyzes the conversion of arginine residues into citrulline (citrullination), a post-translational modification that reduces the positive charge of histones, thereby weakening histone–DNA interactions and leading to alterations in chromatin structure. NET formation and NETosis in ischemic stroke are driven by contribution of DAMPs, platelet-derived signals, coagulation factors, complement activation, inflammatory cytokines and hypoxic stress, all of which trigger PADI4-mediated histone citrullination [74]. Moreover, these epigenetically regulated changes are essential to nuclear envelope rupture and extrusion of DNA–histone–protease complexes during NETosis. Persistent activation of these triggers in the ischemic milieu sustains PADI4 activity, leading to excessive NET formation and prolonged exposure of citrullinated histones and proteases at the blood–brain barrier interface. This integrated triggering network links ischemic tissue injury to epigenetically regulated chromatin remodeling in neutrophils and subsequent BBB disruption. Thus, PADI4-mediated histone citrullination represents a central epigenetic mechanism through which upstream calcium-dependent inflammatory signaling interfaces with chromatin remodeling, transcriptional reprogramming and NET formation-associated pathophysiology [75]. Targeting upstream triggers or PADI4-dependent citrullination may therefore represent a strategic approach to limiting NET-driven neurovascular injury in ischemic stroke.

### 8.2. Acetylation

Beyond the structural effects of histone modification by citrullination, there are other mechanisms. For instance, histone acetylation also contributes to histone unfolding, thereby reshaping transcriptional accessibility and gene regulatory landscapes. Acetylation of histone involves the enzymatic transfer of an acetyl group from acetyl-CoA to lysine residues on histone tails by histone acetyltransferases (HATs), resulting in neutralization of the positive charge on histones and the weakening of histone–DNA interactions [76]. From the perspective of NETosis, histone acetylation is predicted to exert a broader and more sustained impact on chromatin relaxation than site-specific modifications such as histone citrullination [77]. Early attempts to interrogate this mechanism using the HAT inhibitor anacardic acid suggested a link between histone acetylation and NET formation; however, interpretation was confounded by extensive off-target effects, including robust induction of intracellular ROS, autophagy, apoptosis and direct antimicrobial activity [78]. Moreover, anacardic acid-induced NETs exhibited bactericidal activity, contrasting with evidence from more recent studies demonstrating that NETs primarily immobilize rather than kill entrapped bacteria. These findings underscore the need for pharmacological tools with minimal pleiotropic effects to accurately define the role of histone acetylation in NETosis.

In experimental studies, inhibition of histone deacetylases (HDACs) with pan-HDAC inhibitors significantly increased levels of acetylated histone H4 (AcH4) in human neutrophils, which in turn enhanced both baseline and stimulus-induced NET formation and NETosis through NOX-dependent and NOX-independent pathways [78]. Importantly, histone acetylation–mediated enhancement of NET formation occurred without detectable changes in reactive oxygen species (ROS) production, indicating that acetylation does not function as an upstream trigger of ROS signaling but instead operates downstream or independently of it. Moreover, this acetylation-driven NETosis was transcription-dependent, suggesting that histone acetylation promotes chromatin accessibility in transcriptionally active regions, thereby facilitating transcription-associated chromatin decondensation and ultimately enabling NET release.

Notably, the effects of histone acetylation on neutrophil fate are highly dose-dependent. While moderate histone hyperacetylation favors NETosis, excessive acetylation—achieved at higher HDAC inhibitor concentrations—suppresses NET formation, increases NOX-derived ROS production and shifts neutrophil death toward apoptosis. This switch is accompanied by caspase-3 activation and mirrors observations in myeloid cell lines, highlighting a finely tuned balance between epigenetic chromatin relaxation and cell death pathways [78]. Collectively, these findings position histone acetylation as a critical epigenetic checkpoint that integrates transcriptional activity, chromatin dynamics and neutrophil fate decisions during NET formation, acting in concert with yet mechanistically distinctly from histone citrullination.

### 8.3. Methylation

At the chemical level, DNA methylation involves the transfer of a methyl group from S-adenosyl-L-methionine (SAM) to the 5-carbon position of cytosine residues within CpGdinucleotides, a reaction catalyzed by DNA methyltransferases (DNMTs) and resulting in the formation of 5-methylcytosine [79]. This covalent modification alters DNA–protein interactions without changing the underlying nucleotide sequence and serves as a fundamental epigenetic mechanism for regulating gene expression. DNA methylation influences chromatin organization by promoting the recruitment of methyl-CpG-binding proteins and transcriptional repressor complexes, thereby limiting transcription factor accessibility and enforcing gene silencing. Emerging evidence suggests a functional crosstalk between DNMT-mediated DNA methylation and PADI4-driven histone citrullination in regulating neutrophil activation and NET formation [80].

While DNMTs establish transcriptional repression or activation programs through cytosine methylation, PADI4 modulates chromatin accessibility by citrullinating arginine residues on histones, thereby antagonizing histone arginine methylation marks. This reciprocal regulation creates a dynamic epigenetic balance that determines chromatin compaction and gene expression states in neutrophils. Under ischemic and inflammatory conditions, altered DNMT activity can reshape methylation landscapes of genes associated with neutrophil activation, inflammatory responsiveness and chromatin accessibility under ischemic conditions, effectively priming neutrophils for activation [46]. Concurrently, sustained calcium influx activates PADI4, leading to widespread histone citrullination and chromatin decondensation. The convergence of DNMT-dependent transcriptional priming and PADI4-mediated structural chromatin remodeling lowers the threshold for NETosis, facilitating rapid nuclear swelling and extracellular DNA release [81]. Such coordinated epigenetic dysregulation amplifies NET-driven endothelial injury and BBB disruption, highlighting DNMT-PADI4 crosstalk as a critical regulatory axis in ischemic stroke pathology. Current evidence linking DNMT-mediated epigenetic priming with NET-associated neurovascular injury remains largely mechanistic and experimental, with limited direct validation in longitudinal clinical stroke cohorts.

### 8.4. Role of Non-Coding RNAs in Regulating NET Formation

Non-coding RNAs (ncRNAs), especially microRNAs (miRNAs) and long non-coding RNAs (lncRNAs), play a significant role in NET formation and NETosis by mediating gene expression and signaling pathways. This non-coding RNA-mediated epigenetic mechanism controls gene expression for NET formation and regulates vital processes involved in several diseases, including cancer growth, immune dysregulation, sepsis and thrombosis, through the processes of apoptosis, necroptosis, pyroptosis and ferroptosis [82].

MicroRNAs have emerged as critical post-transcriptional regulators of neutrophil activation and NET formation through the fine-tuning of signaling pathways that control chromatin remodeling, reactive oxygen species (ROS) generation and inflammatory gene expression [26]. Several miRNAs have been implicated in modulating NETosis, including miR-146a, miR-155, miR-223, miR-142-3p, miR-21 and miR-9. miR-146a acts as a negative regulator of excessive inflammatory signaling by targeting components of the TLR–NF-κB axis, thereby influencing neutrophil priming and limiting uncontrolled NET release. In contrast, miR-155 promotes proinflammatory neutrophil responses and has been associated with enhanced NET formation through amplification of cytokine signaling and oxidative pathways [27]. miR-223, a key neutrophil-enriched miRNA, regulates granulocyte differentiation and suppresses excessive neutrophil activation; its dysregulation has been linked to increased NETosis and endothelial injury [83]. Additional miRNAs such as miR-142-3p and miR-21 modulate calcium signaling, cytoskeletal dynamics and redox balance, indirectly shaping PADI4 activation and histone citrullination [84]. Collectively, these miRNAs function as epigenetic rheostats that control the threshold and magnitude of NETosis, thereby influencing the extent of NET-mediated blood–brain barrier disruption under ischemic and inflammatory conditions.

lncRNAs contribute to NET formation and NETosis by orchestrating transcriptional priming, chromatin remodeling and inflammatory signaling in activated neutrophils. Nuclear-enriched lncRNAs such as NEAT1 and MALAT1 are upregulated under inflammatory and hypoxic conditions and function as scaffolds for chromatin-modifying enzymes, facilitating histone modifications including citrullination and acetylation, which promote large-scale chromatin decondensation [85].

lncRNAs such as MALAT1, NEAT1, HOTAIR, GAS5 and LINC00520 have been reported to influence neutrophil activation and innate immune responses relevant to NETosis [85,86]. NEAT1 is particularly notable for its role in organizing nuclear paraspeckles and regulating inflammatory gene expression; its upregulation has been associated with enhanced neutrophil activation and sustained inflammatory responses that favor NET release [85,87]. MALAT1 modulates chromatin accessibility and transcriptional elongation, potentially facilitating the transcription-dependent chromatin decondensation required for NET extrusion. Other lncRNAs, including GAS5 and HOTAIR, interact with epigenetic modifiers such as histone methyltransferases and deacetylases, thereby indirectly shaping histone modification landscapes that intersect with PADI4-mediated citrullination [80]. By acting as molecular scaffolds, decoys, or competing endogenous RNAs, lncRNAs integrate inflammatory cues, metabolic stress and epigenetic remodeling to regulate neutrophil fate decisions. Dysregulation of these lncRNAs can therefore amplify NET-driven endothelial damage and BBB permeability, positioning lncRNAs as emerging epigenetic determinants of NET formation or NETosis-associated neurovascular injury. Several lncRNA-mediated regulatory pathways discussed here are supported predominantly by preclinical or indirect evidence, and their precise contribution to BBB dysfunction in ischemic stroke remains to be fully established.

## 9. Epigenetically Regulated NETs as Disruptors of the BBB

**Figure 3:**
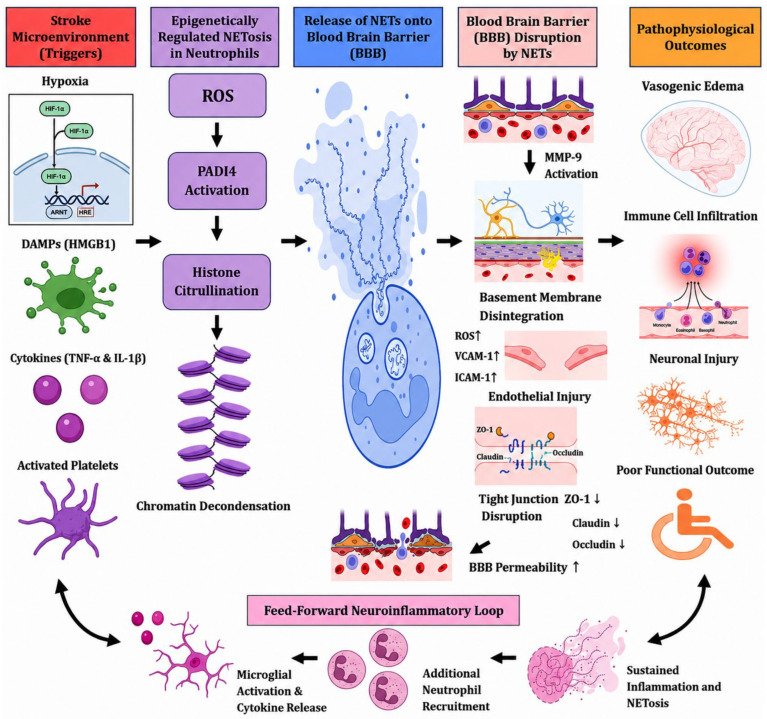
Epigenetically regulated NETosis drives BBB disruption and neurovascular dysfunction in ischemic stroke. Ischemic stroke associated stimuli, including hypoxia, danger-associated molecular patterns (DAMPs), proinflammatory cytokines, and platelet activation, trigger neutrophil activation. This leads to reactive oxygen species (ROS) generation and activation of Peptidyl Arginine Deiminase 4 (PADI4), resulting in histone citrullination (CitH3) and chromatin decondensation and culminating in NET release. NETs, composed of extracellular DNA decorated with myeloperoxidase (MPO) and neutrophil elastase (NE), interact with the neurovascular unit to induce endothelial injury. These effects include disruption of tight junction proteins (claudin-5, occludin, and ZO-1), activation of matrix metalloproteinases (MMP-9), and degradation of the basement membrane, leading to increased blood–brain barrier permeability. The resulting vascular leakage promotes immune cell infiltration, vasogenic edema, and neuronal injury. A feed-forward neuroinflammatory loop involving microglial activation and continued neutrophil recruitment further amplifies NETosis and neurovascular dysfunction.

NETs are now well recognized as critical mediators of thromboinflammation, endothelial injury and secondary tissue damage in ischemic stroke, with substantial evidence implicating NET components in BBB breakdown. Parallel advances have firmly established the roles of inflammation, oxidative stress, platelet–neutrophil interactions and coagulation pathways in driving NETosis and neurovascular dysfunction [20,88]. However, despite growing appreciation that NET formation is fundamentally dependent on chromatin remodeling, the contribution of epigenetic regulation to NET-mediated BBB disruption remains insufficiently explored. In particular, how epigenetically programmed NETs sustain endothelial damage, destabilize tight junction architecture and perpetuate BBB permeability in ischemic conditions has not been investigated in depth. Addressing this gap is essential to understanding how neutrophil-intrinsic chromatin dynamics translate ischemic signals into persistent neurovascular injury. How epigenetically regulated NETosis drives BBB disruption and Neurovascular dysfunction in ischemic stroke is explained in Figure 3.

### 9.1. NET-Induced Endothelial Injury and Tight Junction Disassembly

NET-derived components directly compromise tight junction integrity by targeting both junctional proteins and the endothelial cytoskeleton. Citrullinated histones, particularly CitH3, exert strong cytotoxic effects on endothelial cells by inducing membrane destabilization, calcium influx and actin cytoskeletal contraction, which mechanically disrupt tight junction complexes [40,75]. NET-associated proteases such as neutrophil elastase and matrix metalloproteinases cleave tight junction proteins, including claudin-5, occludin and zonula occludens-1, while simultaneously degrading basement membrane components that provide structural support to the endothelium. In parallel, myeloperoxidase-derived reactive oxidant species oxidize junctional proteins and lipid membranes, further weakening inter-endothelial contacts [42]. Together, these biochemical and proteolytic actions directly dismantle tight junction architecture and initiate BBB leakage [40,42,75]. At the molecular level, NET components activate intracellular signaling pathways that exacerbate endothelial barrier dysfunction. Citrullinated histones and extracellular DNA engage endothelial pattern-recognition receptors, leading to activation of NF-κB and MAPK signaling cascades, which promote inflammatory gene expression and junctional destabilization [72]. These signals induce phosphorylation and internalization of tight junction proteins, reducing their surface expression and impairing junctional reassembly.

NET-associated proteases and inflammatory mediators directly compromise BBB integrity through degradation of tight junction proteins and basement membrane components. Brain microvascular endothelial cells (BMECs) primarily depend on Claudin-5 for maintenance of BBB selectivity, and accumulating evidence suggests that NET-derived proteases including neutrophil elastase, MPO and matrix metalloproteinases promote degradation and mislocalization of claudin-5, whereas evidence regarding claudin-3 remains comparatively limited [89,90]. In addition, NET-associated enzymes degrade extracellular matrix proteins such as laminin and fibronectin, thereby destabilizing the neurovascular unit and exacerbating vascular leakage [91]. Recent studies further indicate that endothelial inflammatory signaling pathways, particularly TLR4/NF-κB activation, contribute to NET-mediated BBB dysfunction by promoting internalization and disruption of tight junction complexes [43,92]. Similarly, RAGE-mediated signaling triggered by NET-associated DAMPs such as HMGB1 has been implicated in cerebrovascular inflammation, although its direct role in cerebral-specific tight junction disruption remains incompletely characterized [93]. Experimental stroke models have additionally associated NET accumulation with reduced claudin-5 and occludin expression and increased BBB permeability [94]. Therefore, while emerging evidence supports brain-specific mechanisms of NET-mediated BBB injury, several receptor-selective and cerebral endothelial pathways still require validation in BMECs and in vivo ischemic stroke models [95,96,97].

In parallel, NET-associated inflammatory signaling may promote epigenetic remodeling within endothelial cells through histone modifications and altered chromatin accessibility affecting genes involved in junctional stability, leukocyte adhesion and inflammatory amplification [53,79]. Such transcriptional reprogramming may further impair endothelial recovery and prolong BBB dysfunction. Concurrently, NET-associated proteases and oxidants promote endothelial apoptosis and senescence, limiting the regenerative capacity of the vascular lining. This coordinated disruption of structural and signaling mechanisms amplifies paracellular permeability and sustains endothelial injury.

Prolonged NET accumulation within the ischemic microvasculature prevents effective restoration of BBB integrity. Persistent exposure to citrullinated histones, proteases and oxidized DNA maintains endothelial activation and suppresses tight junction protein re-expression, thereby prolonging barrier dysfunction. In the cerebral context, this results in continuous plasma protein extravasation, immune cell infiltration and amplification of neuroinflammatory responses [37]. Ultimately, NET-induced tight junction disassembly contributes to vasogenic edema, hemorrhagic transformation and long-term neurovascular instability following ischemic stroke [37,40,42,72,75]. While NET components influence the direct structural and molecular disruption of endothelial tight junction architecture, the broader inflammatory amplification and sustained neurovascular feedback mechanisms contribute to persistent BBB permeability, which is discussed in Section 8.3.

### 9.2. Basement Membrane Degradation and Protease-Mediated Damage

Basement membrane integrity is essential to maintaining vascular stability and BBB function. During ischemic and inflammatory conditions, NETs contribute to basement membrane degradation through the release of proteolytic enzymes and matrix-degrading factors. NET-associated proteases, including neutrophil elastase and matrix metalloproteinases, cleave key basement membrane components such as laminin and type IV collagen, leading to loss of matrix continuity and the weakening of endothelial anchorage. This protease-mediated degradation disrupts endothelial–matrix interactions, promotes detachment of endothelial cells and facilitates increased vascular permeability. Similar mechanisms of basement membrane breakdown have been described in tissue injury and inflammatory models, where excessive protease activity impairs barrier repair and enhances leukocyte transmigration [98,99].

Sustained NET accumulation further amplifies basement membrane damage by perpetuating local inflammatory signaling and secondary protease activation within the neurovascular unit. The proteolytic remodeling of the basement membrane not only enables immune cell infiltration but also compromises vascular repair by disrupting matrix cues required for endothelial regeneration [72,100]. In the context of ischemic stroke, persistent degradation of the basement membrane synergizes with tight junction disassembly to produce prolonged BBB leakage, vasogenic edema and secondary neuroinflammatory injury [101]. These findings position NET-driven protease-mediated basement membrane degradation as a critical structural mechanism underlying sustained BBB dysfunction and neurovascular instability.

### 9.3. NET-Driven Neurovascular Inflammation and BBB Permeability

**Figure 4:**
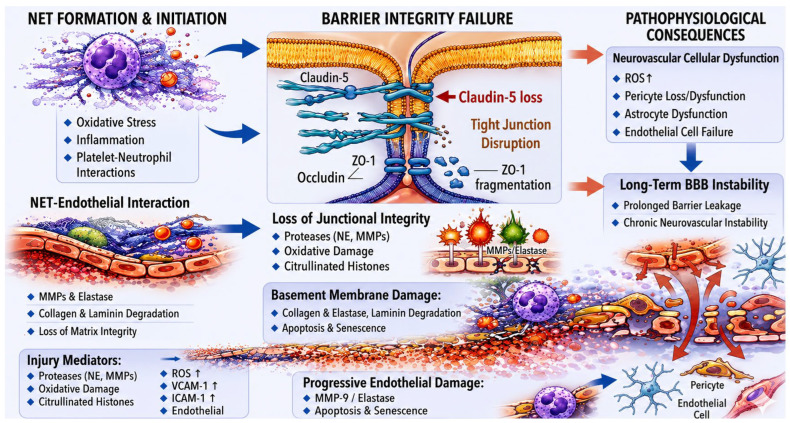
Mechanistic pathways of neutrophil extracellular trap (NET)-mediated disruption of the blood–brain barrier (BBB) in ischemic stroke. The schematic illustrates the sequence of molecular and cellular events leading from neutrophil activation to chronic neurovascular instability. NET formation and initiation are driven by systemic inflammation and platelet–neutrophil crosstalk, promoting the release of chromatin filaments and associated cytotoxic proteins. Upon NET–endothelial interaction, the localized concentration of matrix metalloproteinases (specifically MMP-9) and neutrophil elastase (NE) facilitates the proteolytic degradation of the vascular basement membrane, targeting structural proteins such as collagen and laminin. A critical feature of this pathology is the loss of junctional integrity, characterized by the biochemical downregulation and fragmentation of the tight junction proteins claudin-5, occludin, and zonula occludens-1 (ZO-1). These alterations result in barrier integrity failure, allowing for paracellular leakage and the induction of endothelial senescence and apoptosis. The cumulative pathophysiological consequences involve the progressive dysfunction of the neurovascular unit (NVU), including pericyte loss and astrocyte reactivity, which ultimately transition the microvasculature into a state of long-term BBB instability and chronic neuroinflammation. ROS: reactive oxygen species; ICAM-1: Intercellular Adhesion Molecule-1; VCAM-1: Vascular Cell Adhesion Molecule-1.

Beyond direct endothelial and tight junction injury, NETs also contribute to sustained neurovascular inflammation through inflammatory feedback loops involving endothelial cells, astrocytes, microglia, infiltrating leukocytes and thromboinflammatory mediators. Neutrophil extracellular traps act as potent amplifiers of neurovascular inflammation once deposited within the ischemic microenvironment. NET-derived DNA, citrullinated histones and granular enzymes function as DAMPs that activate endothelial cells as well as perivascular immune cells, including microglia and astrocytes [102]. Engagement of endothelial pattern-recognition receptors by NET components induces NF-κB-dependent transcriptional programs, resulting in increased expression of adhesion molecules, chemokines and proinflammatory cytokines, which promote leukocyte recruitment to the cerebral vasculature [102,103]. This sustained endothelial activation converts transient ischemic insult into a persistent inflammatory state at the BBB interface. Persistent exposure to NET-associated inflammatory mediators may additionally influence chromatin accessibility and transcriptional regulation within endothelial and neurovascular unit cells, thereby sustaining inflammatory signaling and prolonged BBB permeability [53,96,104]. NETs further sustain BBB permeability by reinforcing inflammatory feedback loops within the neurovascular unit. NET-induced endothelial activation promotes secondary release of cytokines and chemokines that activate astrocytes and microglia, which in turn produce reactive oxygen species, nitric oxide and additional inflammatory mediators that exacerbate endothelial injury [104]. This persistent inflammatory crosstalk suppresses endothelial repair programs, delays reassembly of tight junction proteins and interferes with basement membrane restoration (Figure 4). Experimental evidence from models of systemic and cerebral inflammation indicates that prolonged exposure to NET-derived mediators is sufficient to maintain BBB leakage even after resolution of the initiating ischemic trigger, supporting a role for NETs in sustaining long-term neurovascular dysfunction [105].

However, the mechanisms linking NETs to BBB dysfunction remain incompletely resolved, and not all studies support a uniformly direct cytotoxic effect. While NET-associated histones, proteases and PADI4 activity have been implicated in endothelial injury and barrier leakage [7,70], some evidence suggests that BBB disruption may also arise indirectly through secondary activation of microglia, STING signaling, or thromboinflammatory cascades rather than direct NET-mediated endothelial toxicity alone [7]. These differing interpretations raise the possibility that NETs function both as direct effectors and amplifiers of broader inflammatory injury, a distinction with important implications for its therapeutic targeting.

### 9.4. Epigenetic Reprogramming of BBB-Resident Cells by NET Components

Inflammatory and epigenetic regulatory mechanisms appear to function in an interconnected manner during NET-mediated BBB dysfunction. Although accumulating evidence supports the involvement of NET-associated inflammatory mediators in BBB dysfunction, several proposed epigenetic mechanisms remain incompletely characterized in the specific context of ischemic stroke [7,35,79]. Therefore, findings derived from related inflammatory and vascular disease models should be interpreted as supportive mechanistic evidence rather than definitive stroke-specific conclusions [100,101,102,103,104,105]. Emerging evidence suggests that NET-associated inflammatory mediators may differentially influence the epigenetic landscape of distinct neurovascular unit (NVU) cell populations, although direct ischemic stroke-specific evidence remains limited [106,107]. Brain microvascular endothelial cells (BMECs) appear particularly susceptible to NET-associated histone-mediated inflammatory signaling, which has been linked to altered chromatin accessibility, NF-κB-dependent transcriptional activation and suppression of tight junction-associated genes [108,109]. In contrast, astrocytes exposed to inflammatory chromatin fragments and extracellular DNA may exhibit sustained activation of cytokine-associated transcriptional programs involving histone acetylation and inflammatory gene priming, potentially contributing to chronic neuroinflammatory amplification [110]. Pericytes, which are highly sensitive to oxidative and inflammatory stress, may undergo distinct epigenetic alterations involving DNA methylation changes and dysregulated extracellular matrix-associated transcriptional pathways that impair vascular stabilization and BBB repair mechanisms [111].

Importantly, these NVU cell populations may also exhibit differential pharmacological responsiveness to NET-targeted or epigenetic interventions. For example, endothelial injury appears linked more closely to protease- and histone-mediated signaling pathways, whereas astrocytic inflammatory activation may be comparatively more sensitive to chromatin-modifying and cytokine-regulatory mechanisms [106,112]. However, the precise cell-specific epigenetic signatures, including differential DNA methylation patterns, histone modification profiles and chromatin remodeling states induced by NET exposure, remain insufficiently characterized in ischemic stroke models and require further experimental validation.

Where direct ischemic stroke-specific neurovascular evidence remains limited, the discussed mechanisms should be interpreted cautiously as supportive or hypothesis-generating observations rather than experimentally confirmed cerebral-specific pathways. Moreover, NET-mediated epigenetic responses may vary across distinct neurovascular unit cell populations, including endothelial cells, astrocytes and pericytes, depending on their inflammatory and metabolic microenvironment [79,100]. Reports have suggested that endothelial cells, astrocytes and pericytes may display distinct epigenetic signatures following NET exposure, including differential patterns of DNA methylation, histone acetylation and inflammatory chromatin accessibility [52,106]. Such cell-type-specific epigenetic remodeling may differentially alter neurovascular unit function within the BBB microenvironment.

NET-derived chromatin, citrullinated histones and associated proteases interact directly with cerebral endothelial cells, astrocytes and pericytes, triggering intracellular signaling pathways that converge on chromatin-modifying enzymes [113]. Exposure of endothelial cells to extracellular histones and DNA has been shown to activate calcium influx, MAPK signaling and NF-κB-dependent transcription, processes that are tightly linked to changes in histone acetylation and methylation patterns governing inflammatory and permeability-associated gene expression [114].

In endothelial cells, NET components promote sustained transcriptional activation of adhesion molecules, chemokines and matrix-degrading enzymes through the epigenetic remodeling of promoter and enhancer regions [115]. Histone modifications such as increased H3 acetylation and altered arginine methylation facilitate prolonged accessibility of genes encoding ICAM-1, VCAM-1, MMP-9 and inflammatory cytokines, thereby reinforcing leukocyte recruitment and barrier destabilization [46].

In endothelial cells, these epigenetic alterations appear to favor a permeability-associated transcriptional phenotype characterized by suppression of junctional repair pathways [103]. In contrast, astrocytes and pericytes may undergo distinct methylation and histone modification responses regulating cytokine secretion, oxidative stress and extracellular matrix remodeling [104]. Endothelial cells appear particularly susceptible to NET-associated chromatin and inflammatory signaling, whereas astrocytes and pericytes may contribute indirectly through secondary cytokine amplification, oxidative stress and neurovascular inflammatory crosstalk [100,104,105].

Astrocytes and perivascular cells are also susceptible to NET-mediated epigenetic reprogramming. NET-derived histones and neutrophil elastase stimulate astrocytic pattern-recognition receptors, resulting in altered DNA methylation and histone modification profiles at loci regulating cytokine production, glutamate homeostasis and vascular support functions [116]. These changes bias astrocytes toward a proinflammatory, barrier-disruptive phenotype characterized by increased secretion of IL-1β, TNF-α and VEGF, factors known to compromise tight junction integrity and endothelial stability [117]. Such epigenetically imprinted astrocytic responses may persist beyond the acute phase of injury, contributing to chronic BBB dysfunction.

Although direct stroke-specific epigenomic evidence in pericytes remains limited, available inflammatory models suggest that NET-associated mediators may alter pericyte chromatin states and transcriptional programs regulating vascular integrity, basement membrane maintenance and endothelial support functions [52,105]. Further cell-type-specific epigenomic studies are required to define whether distinct DNA methylation and histone modification profiles emerge across BBB-resident cells following NET exposure.

Collectively, NET-induced epigenetic alterations across BBB-resident cell populations create a feed-forward loop in which chromatin reprogramming sustains inflammatory gene expression, suppresses barrier-reparative pathways and amplifies neurovascular permeability [45,113,114,115]. By acting simultaneously on neutrophils and BBB cells, NETs establish an epigenetically reinforced inflammatory niche that favors persistent vascular leakage and secondary neuronal injury in ischemic stroke. This emerging paradigm positions NET components as transcellular epigenetic modifiers within the neurovascular unit, extending their pathological influence beyond acute structural damage to long-term regulation of BBB function. A summary of the major NET components, their target BBB-resident cells, associated epigenetic modifications, and functional consequences is provided in Table 2.

## 10. Therapeutic Targeting of Epigenetic NET Formation–BBB Axis in Ischemic Stroke

Targeting NET formation and its epigenetic regulation to preserve BBB integrity and limit neurovascular injury following ischemic stroke remains underexplored. NETosis is fundamentally dependent on chromatin remodeling, histone post-translational modifications and transcriptional reprogramming. Therapeutic interventions aimed at epigenetic regulators of the NET formation–BBB axis could be implemented through a phased or step-wise approach that includes inhibition of key chromatin-modifying enzymes, modulation of DNA methylation and histone acetylation pathways, the targeting of the non-coding RNA network, and direct degradation of NET components. Such a temporally guided therapeutic strategy may be particularly important in ischemic stroke, where the mechanisms driving NET-mediated BBB dysfunction evolve dynamically across the early, subacute and chronic phases of injury. During the early phase, therapeutic approaches that limit neutrophil recruitment, platelet–neutrophil interactions, endothelial activation and initial thromboinflammatory signaling may help attenuate early vascular injury and inflammatory amplification. In the subacute phase, when NET burden, PADI4 activation, histone citrullination, protease release and thromboinflammation reach maximal intensity, targeted inhibition of NET formation, extracellular proteases and chromatin remodeling pathways may provide greater protection against BBB disruption. In the chronic phase, persistent inflammatory signaling and epigenetic reprogramming may sustain neurovascular dysfunction even after the initial ischemic insult; therefore, therapeutic modulation of DNA methylation, histone acetylation and non-coding RNA regulatory networks may help promote BBB restoration, vascular remodeling and long-term neurological recovery.

The subsequent sections further elaborate these phase-specific therapeutic approaches, including inhibition of PADI4-driven histone citrullination, modulation of DNA methylation and histone acetylation pathways, the targeting of non-coding RNA networks, and degradation or neutralization of NET components. Nevertheless, the therapeutic targeting of the NETosis–BBB axis requires careful temporal consideration, as NETs may also contribute to host defense, immune surveillance and post-ischemic tissue remodeling. Consequently, indiscriminate or sustained suppression of NET formation could potentially impair reparative neurovascular responses. Collectively, these observations underscore the importance of a temporally selective and mechanistically tailored therapeutic strategy in which the efficacy and safety of NET-targeted interventions are likely to depend on the evolving stage of ischemic injury and BBB dysfunction.

### 10.1. PADI4 Inhibition as a Central Strategy

PADI4 is a key enzyme which drives NET formation through histone citrullination, and experimental studies have consistently demonstrated that PADI4 activity is indispensable to NET formation [60,126]. For instance, PADI4-mediated histone hypercitrullination induces large-scale chromatin unfolding, enabling extracellular release of DNA–histone complexes characteristic of NETs [60]. Mechanistically, citrullination antagonizes histone arginine methylation and disrupts chromatin compaction, thereby facilitating transcriptional activation and nuclear expansion [126]. In vivo evidence further supports the pathogenic role of PADI4-driven NETosis in vascular dysfunction. Genetic deletion of PADI4 significantly attenuates endothelial dysfunction and reduces vascular inflammation, highlighting its importance in NET-mediated endothelial injury [127]. These findings are particularly relevant to BBB disruption, where endothelial integrity is critically compromised.

Pharmacological inhibition of PADI4 using agents such as Cl-amidine has shown efficacy in reducing NET formation in preclinical models. Emerging studies on natural and synthetic PADI4 inhibitors further suggest that targeting this enzyme can effectively suppress pathological NETosis without completely impairing host defense mechanisms [128]. Collectively, these observations position PADI4 as a master epigenetic regulator of NET formation and a highly attractive therapeutic target for mitigating BBB disruption in ischemic stroke.

### 10.2. Targeting Histone Acetylation Pathways

Histone acetylation represents another critical epigenetic mechanism regulating NET formation [76]. This process enhances transcriptional accessibility and facilitates chromatin decondensation required for NET release.

Evidence indicates that histone acetylation exerts a biphasic effect on NETosis. Moderate levels of acetylation promote NET formation, whereas excessive acetylation shifts neutrophil fate toward apoptosis rather than NETosis [77]. Histone deacetylase (HDAC) inhibitors, therefore, exhibit context-dependent effects. Although certain HDAC inhibitors enhance NETosis by promoting chromatin accessibility, higher concentrations suppress NET formation and induce apoptotic pathways.

This duality has important therapeutic implications. Selective modulation of histone acetylation, rather than complete inhibition, may allow for the fine-tuning of neutrophil responses to limit pathological NET formation without compromising immune function [129]. Moreover, HDAC inhibitors may exert additional protective effects on BBB integrity by modulating endothelial inflammation and oxidative stress.

### 10.3. DNA Methylation and DNMT Targeting

DNA methylation, mediated by DNA methyltransferases (DNMTs), plays a crucial role in regulating gene expression associated with neutrophil activation and NETosis [79]. Methylation of CpG islands typically represses gene transcription by restricting transcription factor access and promoting chromatin condensation.

Emerging evidence suggests that DNA methylation interacts dynamically with histone modifications during NETosis [80]. DNMT-mediated transcriptional priming can regulate genes involved in calcium signaling, oxidative stress and inflammatory responses, thereby lowering the threshold for NET formation. Simultaneously, PADI4-mediated citrullination antagonizes histone methylation marks, creating a permissive chromatin environment for NETosis [130,131].

Pharmacological DNMT inhibitors such as decitabine and azacitidine have been shown to modulate inflammatory gene expression and chromatin accessibility [132]. Although their role in NETosis remains underexplored, these agents may indirectly suppress NET formation by altering transcriptional programs in neutrophils. However, their clinical application in stroke is limited by potential off-target effects and systemic immunosuppression.

### 10.4. Non-Coding RNA-Based Therapeutic Strategies

Non-coding RNAs (ncRNAs), including microRNAs (miRNAs) and long non-coding RNAs (lncRNAs), serve as critical regulators of gene expression and chromatin dynamics in neutrophils [132,133]. These molecules modulate key signaling pathways involved in NETosis, including calcium signaling, ROS production and inflammatory transcriptional networks.

Specific miRNAs such as miR-146a, miR-155 and miR-223 have been implicated in regulating neutrophil activation thresholds and NET formation [134]. For instance, miR-146a acts as a negative regulator of inflammatory signaling, whereas miR-155 promotes proinflammatory responses and enhances NETosis. Dysregulation of these miRNAs contributes to excessive NET formation and endothelial injury.

Therapeutically, miRNA mimics or inhibitors (antagomirs) offer a targeted approach to modulate NETosis at the post-transcriptional level. Similarly, lncRNAs such as NEAT1 and MALAT1 regulate chromatin accessibility and inflammatory gene expression, providing additional therapeutic targets [135]. Although still in early stages, ncRNA-based therapies hold promise for precision modulation of epigenetic pathways in NETosis and BBB dysfunction.

### 10.5. NET Degradation and Neutralization Strategies in the NET Formation–BBB Axis

Beyond targeting upstream epigenetic regulators of neutrophil extracellular trap (NET) formation, direct degradation and neutralization of NET structures represent an effective and mechanistically distinct therapeutic strategy to mitigate BBB disruption in ischemic stroke. One of the most extensively investigated strategies involves the use of DNase I, which enzymatically cleaves extracellular DNA and disrupts the structural scaffold of NETs [136]. The presence of NETs within thrombi and cerebral vasculature has been clearly demonstrated in ischemic stroke, where they contribute to thrombus stability and resistance to thrombolysis. Importantly, NET-rich thrombi exhibit reduced susceptibility to tissue plasminogen activator (tPA), and DNase I has been shown to enhance thrombolytic efficacy by degrading the DNA backbone of NETs [16]. Subsequent studies further confirmed that NET degradation improves clot permeability and facilitates fibrinolysis, highlighting its therapeutic relevance in acute stroke management [18].

Beyond DNase-based approaches, neutralization of specific NET components represents another important therapeutic strategy [72]. Extracellular histones, particularly H3 and H4, are among the most cytotoxic components of NETs and have been shown to directly damage endothelial cells and increase vascular permeability. Neutralizing antibodies against histones significantly reduces endothelial injury and inflammatory responses in vivo, suggesting a protective role in vascular integrity [137]. Given the mechanistic overlap between systemic endothelial injury and BBB disruption, histone neutralization may represent a promising strategy to limit NET-induced neurovascular damage.

Another critical target is NE, a key enzyme involved in both NET formation and extracellular matrix degradation. During NETosis, NE translocates to the nucleus and facilitates chromatin decondensation; once released extracellularly, it degrades tight junction proteins and basement membrane components. Inhibition of neutrophil elastase has been shown to reduce tissue injury and vascular permeability in inflammatory models [138]. Similarly, MMPs, particularly MMP-9, play a crucial role in BBB disruption by degrading extracellular matrix components and tight junction proteins [139]. Elevated MMP-9 levels are strongly associated with BBB breakdown and hemorrhagic transformation in stroke, and their inhibition has been shown to preserve BBB integrity [139,140].

Emerging evidence also highlights the importance of NET–platelet interactions in stabilizing NET structures and amplifying their pathological effects. Platelets bind to NETs through interactions involving P-selectin and platelet factor 4 (PF4), enhancing thrombus formation and protecting NETs from degradation [141]. Disruption of platelet–NET interactions has been shown to reduce thrombosis and vascular inflammation, suggesting an additional therapeutic avenue [142].

Importantly, while NET degradation and neutralization strategies show considerable promise, complete elimination of NETs may not be desirable due to their role in host defense. NETs are essential to trapping and neutralizing pathogens and excessive suppression could increase susceptibility to infections. Therefore, therapeutic approaches should aim for controlled modulation rather than complete inhibition of NET activity. These considerations further emphasize that therapeutic modulation of NETosis must balance neurovascular protection with preservation of physiological immune defense and tissue repair mechanisms. Additional limitations should also be considered when interpreting the therapeutic potential of NET-targeted interventions. DNase-based strategies may degrade extracellular NET scaffolds without fully neutralizing associated histones, proteases and inflammatory mediators that continue to exert endothelial toxicity [143]. Similarly, prolonged inhibition of PADI4, neutrophil elastase or epigenetic regulators may produce unintended systemic immunological effects because these pathways participate in broader host defense and inflammatory signaling networks beyond NETosis. Variability in stroke timing, thrombus composition and patient-specific inflammatory responses may further influence therapeutic efficacy and complicate optimal treatment stratification. Moreover, most currently available evidence remains derived from experimental or ex vivo studies, while stroke-specific clinical validation and long-term safety data remain limited.

In the context of the NETosis–BBB axis, combining NET degradation strategies with epigenetic interventions may provide synergistic benefits. Epigenetic therapies targeting PADI4, DNMTs, or histone acetylation can reduce NET formation at the source, while DNase-based approaches eliminate already-formed NETs. Such combined strategies may effectively disrupt the feed-forward cycle of NET-induced endothelial injury, inflammation and BBB permeability.

### 10.6. Combination and Multi-Target Therapeutic Approaches

Given the interconnected nature of NETosis, epigenetic regulation and BBB dysfunction, single-target interventions may have limited efficacy, whereas multi-target therapeutic strategies may offer greater potential to disrupt pathogenic feed-forward loops sustaining neurovascular injury in ischemic stroke [10,16]. Combination approaches targeting complementary nodes within these pathways may therefore provide superior therapeutic benefit.

Co-targeting PADI4-mediated NET formation with DNase I-mediated NET degradation represents one promising strategy, as such an approach may simultaneously suppress NET release and promote clearance of extracellular chromatin scaffolds that contribute to thromboinflammation and BBB injury [16,18]. Likewise, combining HDAC modulators with anti-inflammatory agents may allow coordinated regulation of chromatin accessibility and inflammatory signaling, thereby attenuating both epigenetically driven neutrophil activation and downstream neurovascular injury [26,27].

Similarly, ncRNA-based interventions used alongside epigenetic inhibitors may offer finer control over transcriptional and post-transcriptional pathways involved in NETosis and endothelial dysfunction. In particular, targeting dysregulated microRNAs such as miR-146a and miR-155 in combination with broader epigenetic modulators may represent a precision-oriented approach to modulating inflammatory circuits relevant to stroke pathology [26,27].

Given the temporal and mechanistic heterogeneity of ischemic injury, combinatorial strategies may also better accommodate phase-specific therapeutic windows and overcome pathway redundancy, which often limits single-agent therapies [25,37]. Such multi-target approaches may therefore be particularly effective in interrupting the self-reinforcing inflammatory and epigenetic circuits that sustain BBB disruption in ischemic stroke and may represent an important future direction for neurovascular protection and precision therapeutics.

## 11. Knowledge Voids, Clinico-Translational Implications and Future Perspectives

Growing evidence supports the clinical relevance of the epigenetic NET formation–BBB axis in ischemic stroke, with circulating NET biomarkers such as CitH3 and MPO-DNA complexes correlating with disease severity, thrombus stability, resistance to thrombolysis, hemorrhagic transformation and vascular injury [11,12,118]. These findings highlight the translational potential of integrating NET biomarkers with epigenetic profiling for patient stratification and targeted therapeutic intervention.

Despite this progress, important knowledge gaps remain. Limited human studies have examined epigenetic regulation of NET formation in stroke, cell-type-specific epigenetic mapping within the neurovascular unit is lacking, and the temporal dynamics of NET-driven BBB disruption remain incompletely understood. In addition, no clinical trials have yet specifically targeted epigenetic NET formation pathways, underscoring major barriers to translation [73]. Interpretation of experimental findings is also limited by the constraints of current stroke models, which may not fully reproduce the temporal, immunological and vascular complexity of human ischemic stroke. Species-specific inflammatory responses, differences in cerebral vascular architecture and variability in NET detection approaches may influence the observed extent and pathological significance of NET formation. Furthermore, the reliable real-time monitoring of NET dynamics within the neurovascular compartment remains technically challenging.

Addressing these gaps will require multidisciplinary and technology-driven approaches, including single-cell epigenomic profiling, multi-omics integration, CRISPR-based epigenetic editing and data-driven discovery using artificial intelligence and machine learning to identify novel biomarkers and therapeutic targets. Such approaches may help refine mechanistic understanding and accelerate precision therapeutic development.

Therefore, epigenetically regulated NET formation represents a central mechanism linking innate immune activation to neurovascular injury, while the epigenetics–NET formation–BBB axis offers a promising framework for biomarker-guided and mechanism-based interventions. Bridging current knowledge gaps through translational research may help advance new strategies for preserving BBB integrity and improving outcomes in ischemic stroke and related neuroinflammatory disorders. Overall, the proposed NET formation–epigenetics–BBB framework should currently be interpreted as an evolving mechanistic model supported by emerging experimental evidence rather than a fully established pathogenic pathway in human ischemic stroke.

## 12. Limitations of This Study

This review has several limitations. The proposed epigenetic NET formation–BBB axis is based largely on emerging evidence and some mechanistic relationships remain incompletely resolved. Many of the available data originate from experimental models, while clinical and longitudinal human studies validating these interactions remain limited. In addition, the absence of cell-type-specific epigenomic profiling data for endothelial cells, astrocytes and pericytes under ischemic conditions restricts the precise understanding of how NET-derived mediators remodel chromatin landscapes across the neurovascular unit. The heterogeneity of ischemic stroke pathophysiology and epigenetic regulation may further restrict generalization of current findings. Finally, several of the therapeutic strategies discussed, including DNMT modulation, histone modification targeting and ncRNA-based interventions, remain largely exploratory and are supported primarily by indirect evidence derived from inflammatory, vascular or non-cerebral disease models, warranting substantial stroke-specific translational validation.

## Figures and Tables

**Table 2:** Mapping of NET components to epigenetic modifications in BBB-resident cells.

NET Component	Primary Target Cells at BBB	Evidence Level	Epigenetic Modifications and Molecular Pathways	Functional Consequences	Authors
Citrullinated Histone H3 (CitH3)	Endothelial cells and astrocytes	Direct Stroke Evidence	Histone arginine citrullination, antagonism of repressive arginine methylation, and increased chromatin accessibility.	Sustained inflammatory gene transcription, tight junction disruption, and endothelial cytotoxicity.	Chen et al., 2025 [118]
Neutrophil Elastase (NE)	Endothelial cells and pericytes	Indirect Related-Model Evidence	Indirect chromatin remodeling via PAR-2 signaling influences histone acetylation through nuclear translocation.	Tight junction protein degradation, impaired endothelial repair, and BBB destabilization.	Zhao et al. 2015, Du et al., 2026 [119,120]
Myeloperoxidase (MPO)	Endothelial cells and astrocytes	Direct Stroke Evidence	Oxidative DNA damage, interference with DNMT binding leading to altered DNA methylation patterns, and chromatin remodeling.	Persistent inflammatory gene activation and suppression of BBB repair pathways.	Chen et al., 2024 [121]
Cell-Free DNA (cfDNA)	Endothelial cells and microglia	Direct Stroke Evidence	Activation of TLR9 and cGAS–STING sensing pathways, secondary histone acetylation, and DNA methylation changes.	Epigenetic priming of inflammation, prolonged BBB permeability, and leukocyte recruitment.	Roth et al., 2023 [122]
NET-Associated Proteases (MMP-9 and Cathepsins)	Endothelial cells	Indirect Related-Model Evidence/Hypothesis	Promoter acetylation/demethylation of MMP genes and epigenetically reinforced protease expression.	Basal lamina degradation, sustained endothelial dysfunction, and vascular leakage.	Gurney et al. 2006 [123]
Extracellular Histones (H3 and H4)	Endothelial cells	Indirect Related-Model Evidence	Induction of histone acetylation changes and chromatin remodeling via Ca^2+^ influx and NF-κB activation.	Endothelial toxicity, increased permeability, and inflammatory amplification.	Villalba et al., 2020 [124]
Platelet–NET complexes (PF4-Bound NETs)	Endothelial cells	Direct Stroke Evidence	Epigenetic activation via NF-κB-mediated histone acetylation and inflammatory signaling.	Enhanced leukocyte adhesion, sustained vascular inflammation, and immunothrombosis.	Mohiuddin et al., 2026 [125]

BBB: blood–brain barrier; Ca^2+^: calcium ion; cGAS-STING: cyclic-GMP-AMP Synthase-Stimulator of interferon genes; DNMT: DNA methyltransferase; MMP-9: matrix metalloproteinase-9; NF-κB: Nuclear factor kappa-light-chain-enhancer of activated B cells; NETs: neutrophil extracellular traps; PAR-2: Protease-activated Receptor-2; PF4: platelet factor 4; TLR9: Toll-like Receptor-9.

## Data Availability

The data used to support the findings of this study are available from the corresponding author upon request.
